# Chronic Constipation Unmasking as Hirschsprung Disease in a Preadolescent: Delayed Presentation or Delayed Diagnosis?

**DOI:** 10.7759/cureus.60315

**Published:** 2024-05-14

**Authors:** Abhilasha Bhargava, Kiran Khedkar

**Affiliations:** 1 General Surgery, Jawaharlal Nehru Medical College, Datta Meghe Institute of Higher Education and Research, Wardha, IND; 2 Pediatric Surgery, All India Institute of Medical Sciences, Nagpur, IND

**Keywords:** late diagnosis, hirschsprung disease, colostomy, chronic constipation, abdominal distension

## Abstract

Hirschsprung disease, a rare genetic disorder affecting the enteric nervous system, is characterized by the absence of ganglion cells in the myenteric plexus. Typically identified in neonates due to the failure to pass meconium, diagnosis beyond the first year of life is considered delayed. Common clinical manifestations in children with late-onset Hirschsprung disease include abdominal distension, abdominal pain, vomiting, fever, and abnormal bowel sounds. Sigmoid volvulus, though uncommon, can complicate Hirschsprung disease, potentially leading to misdiagnosis and severe complications such as intestinal perforation, hemorrhage, sepsis, and even mortality. Non-surgical interventions such as antibiotic therapy, intestinal decompression, and fluid resuscitation are preferred initial treatments to stabilize the patient. This case involves a 9-year-old boy who has presented with abdominal distension since birth and a lengthy history of irregular bowel habits. The diagnosis of Hirschsprung disease was confirmed at our institution, and the patient underwent a two-stage repair procedure, which was completed without any intraoperative or postoperative complications. The patient experienced an uneventful recovery, was discharged with stable vital signs, and regained normal bowel function. This case highlights the challenges of delayed diagnosis at nine years and underscores the importance of prompt management.

## Introduction

Hirschsprung disease is a disorder of the enteric nervous system characterized by the absence of ganglia in the intestinal plexuses. It is a rare genetic condition, with an incidence rate typically around one out of every 5000 individuals [1]. Familial and isolated forms of Hirschsprung disease have been associated with various genetic factors, although mutations in the *EDNRB* and *RET* proto-oncogenes are commonly reported. Mutations on chromosome 10q were among the first identified as causative in Hirschsprung disease [2], with certain mutations in the *RET* gene impacting *SOX10* binding and correlating with a four-fold increase in incidence rates [3]. Diagnosis of Hirschsprung disease commonly occurs in neonates, often identified by the characteristic inability to pass meconium within the first days of life [4,5]. When diagnosis is delayed until after the first year of life, it is considered a late diagnosis, which is rare but associated with an increased risk of complications later in life, although the literature presents mixed evidence [6]. This case involves a 9-year-old boy admitted with abdominal distension, ultimately diagnosed with Hirschsprung disease. The child had a late diagnosis of Hirschsprung disease due to a lack of awareness and limited medical facilities. He underwent surgical management with a two-stage repair procedure. Follow-up at one, three, and six months postoperatively showed a successful recovery, with no new complaints reported. A second-stage repair was performed nine months later, and the patient experienced an uneventful recovery period, regaining normal bowel function after the second surgery.

## Case presentation

**Figure 1:**
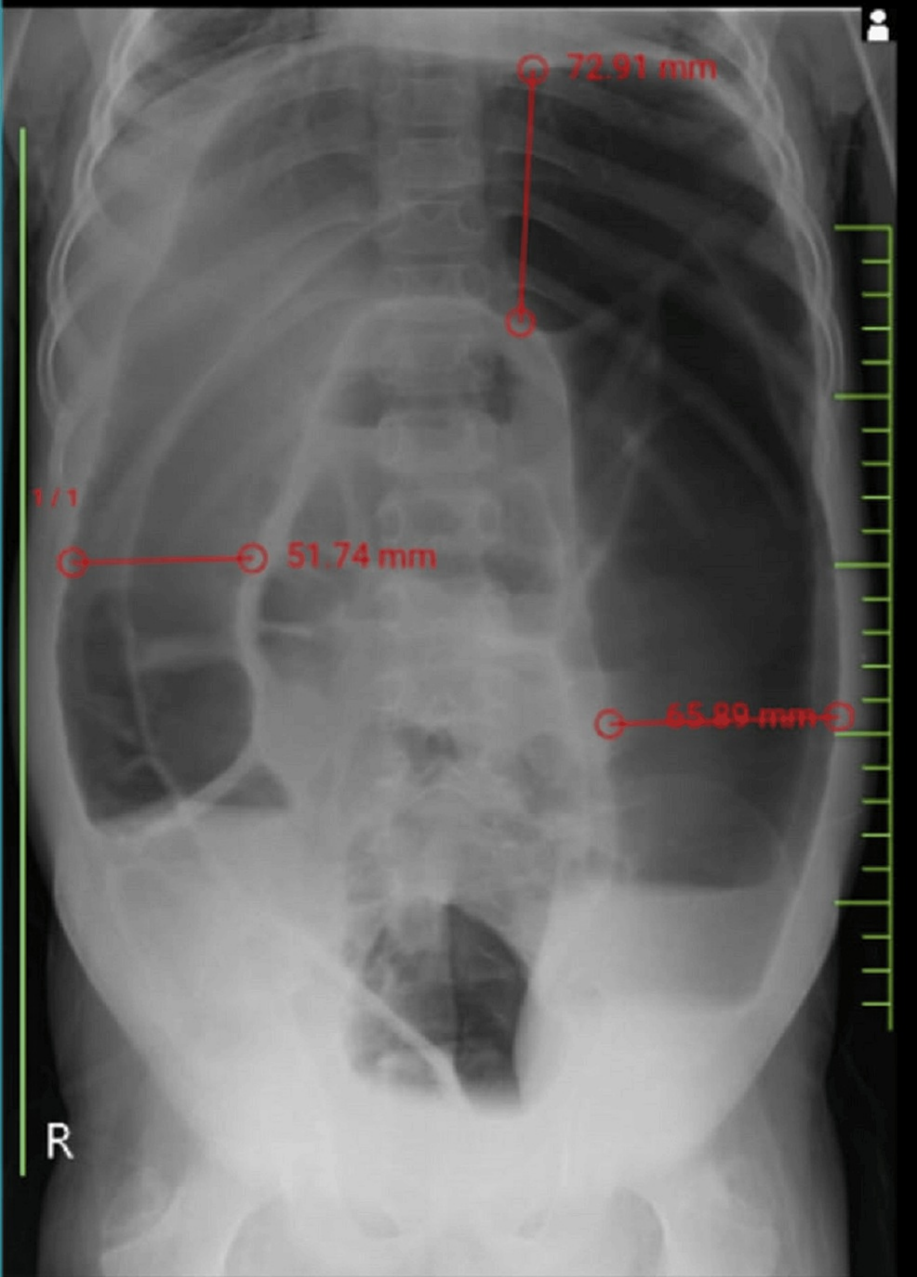
Abdominal radiograph showing dilated large bowel loops, with the largest dimension in the transverse colon noted as 72.91 mm

**Figure 2:**
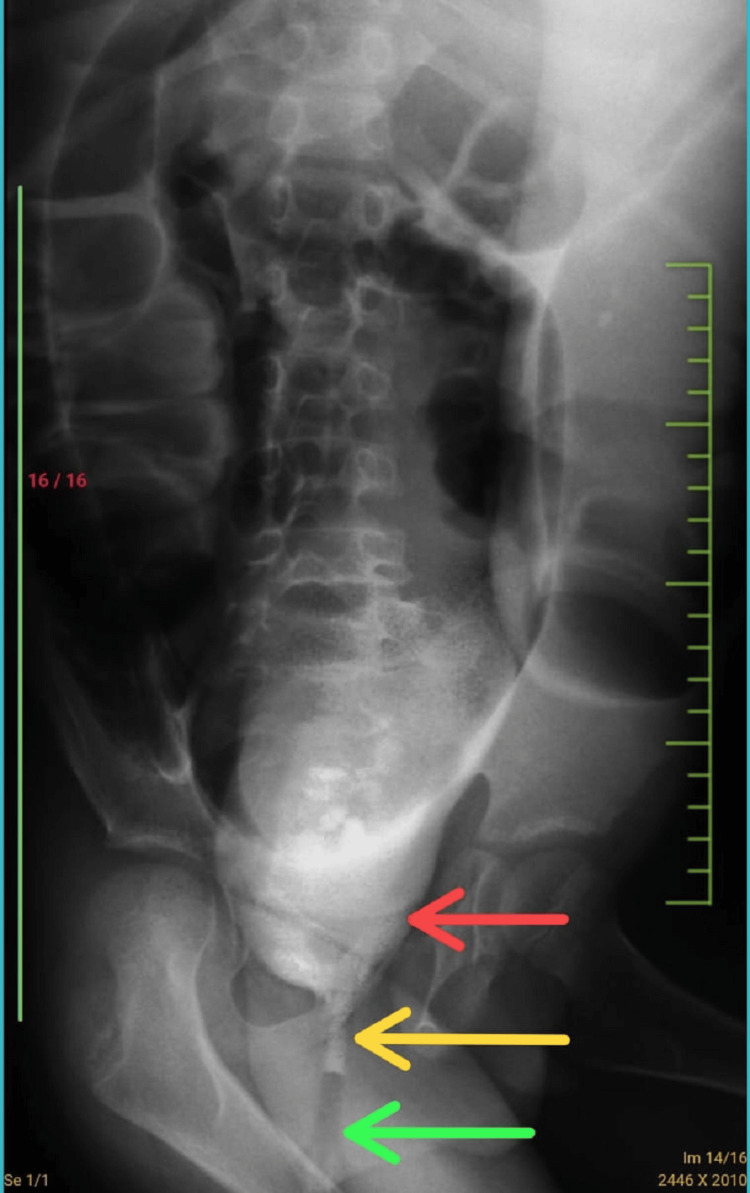
Contrast enema showing the transition zone The red arrow shows a dilated colon, the yellow arrow shows the transition zone, and the green arrow shows a narrow rectum.

A 9-year-old male, weighing 25 kg and measuring 126 cm in height, was admitted to our hospital with a complaint of chronic constipation and abdominal distension since birth, with the passage of stools every third day as reported by the parents. Abdominal distention was insidious in onset and used to resolve on its own. With the current presentation, the distension gradually progressed to the present size, associated with the passage of foul-smelling, watery, loose stools every third to fourth day for the past 15 days. The patient visited a local hospital, where he underwent an abdominal ultrasound, which was suggestive of subacute obstruction. The patient was diagnosed with paralytic ileus and was referred to our tertiary care hospital for further management. On admission, the patient was vitally stable. The patient was advised rectal washes and was prescribed intravenous injections of metronidazole. For further evaluation, he was advised a contrast enema, which was suggestive of evidence of dilated large bowel loops with a transitional zone. This brought us closer to the diagnosis of Hirschsprung disease (Figures 1, 2).

**Figure 3:**
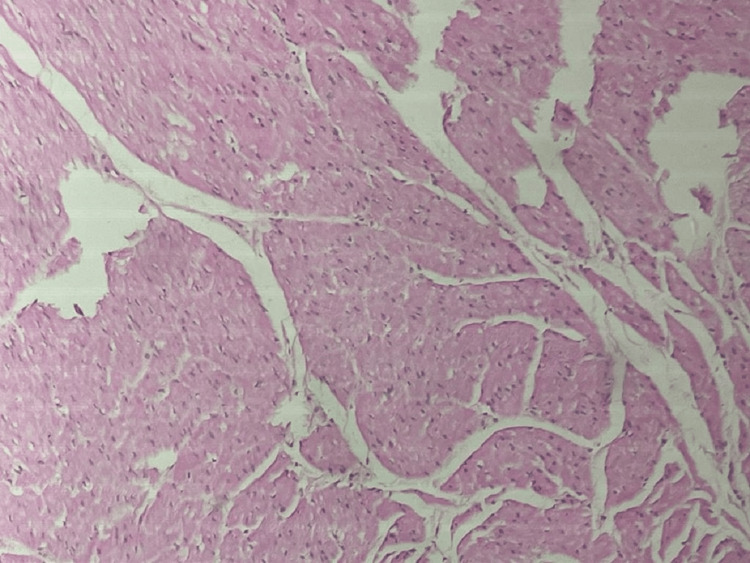
Histopathology slide showing Hirschsprung disease—the absence of ganglion cells and hypertrophic nerve bundles

The patient was planned for leveling colostomy, and intraoperatively, there was evidence of an edematous, grossly dilated large intestine that was thickened up to the distal descending colon. He was subjected to multiple seromuscular biopsies from the rectum and the suspected transitional zone, which were sent for an intraoperative frozen section examination. Excised sections showed the absence of ganglion cells in the biopsy from the rectum and the presence of ganglion cells in the proximal dilated bowel (Figure 3).

Multiple seromuscular biopsies were taken during surgery and sent for a frozen section examination, and the site where ganglion cells are seen on the frozen section was selected for colostomy. Subsequently, the left descending stoma was performed and fixed. The patient started passing stools on the second postoperative day. Hematoxylin and eosin (H&E) staining confirmed the frozen section findings. The patient regularly passed stools and flatus from the colostomy site, and abdominal distension subsided. The patient was again admitted after nine months for second-stage repair by the Soave procedure. Following preoperative investigations, he was taken up for a thorough procedure. The patient passed stools and flatus on postoperative day three. The patient was followed up after three months with no other complaints at follow-up. The patient developed adhesive bowel obstruction after three months, which was managed by laparotomy and adhesiolysis.

## Discussion

Hirschsprung disease is typically diagnosed during the neonatal period, and its recognition after the first year of life is rare in medical literature. Pediatricians may face challenges in diagnosing Hirschsprung disease, as fewer than 10% of cases present with clinical symptoms, often resulting in delayed referrals and a lack of apparent clinical indicators [5,6]. In rare instances, Hirschsprung disease may present concurrently with sigmoid volvulus, leading to acute intestinal obstruction [7-9]. Clinical presentations in affected children commonly include abdominal distension, abdominal pain, vomiting, fever, and abnormal bowel sounds [9,10]. Diagnosis can be achieved through various methods, such as radiological imaging, contrast enemas, and anorectal manometry, with histopathological examination providing confirmatory evidence [11]. To supplement the diagnosis, the patient underwent a biopsy of the suspected transitional zone since the sensitivity of contrast enema has been reported at 62.5% [12].

Immunohistochemistry, particularly calretinin and acetylcholinesterase staining, is crucial in the diagnosis of Hirschsprung disease in children and neonates, as it aids in identifying immature ganglionic cells within the submucosal and myenteric plexuses [13,14]. Due to the lack of facilities, anal manometry was not conducted at our tertiary care center. Differential diagnosis for chronic constipation in children encompasses a range of conditions, including Hirschsprung disease, congenital anorectal malformations, neurologic disorders, and metabolic causes. Radiological findings in Hirschsprung disease often reveal a dilated proximal colon, narrowed rectum, and retention of stool and barium after enema administration [15]. Toxic megacolon with colonic dilation and mucosal edema can also manifest clinically. The length of the transition zone, typically located proximally in the colon and consisting of aganglionic regions, is variable but often measures 3-5 cm [14]. While non-surgical interventions have been explored as primary approaches for late-onset Hirschsprung disease, such as saline enemas for intestinal decompression, surgical management remains the definitive treatment [16]. Radiological imaging commonly reveals intestinal distention in sigmoid loops, indicative of acute obstruction.

In our case, no pediatric surgical services were available at a nearby hospital. Since the patient's parents were financially weak, they could not afford to go to a distant hospital. These factors culminated in the patient's late diagnosis. Such delays in diagnosis can be prevented by routine screening of the general public in the form of health camps. Government schemes can help with free medical checkups in affiliation with tertiary health centers [17]. Clinical management decisions in cases of late-diagnosed Hirschsprung disease should prioritize ruling out the condition through various diagnostic modalities to avoid adverse outcomes [6,8,16,18]. A timely and accurate diagnosis is paramount for reducing morbidity and mortality through prompt intervention.

## Conclusions

In this case, the diagnosis of Hirschsprung disease was not delayed due to a late presentation of symptoms but rather due to the unavailability of pediatric surgery services. Late diagnosis of Hirschsprung disease in pediatric cases has been associated with adverse health effects. Delays or misdiagnosis can lead to complications such as intestinal perforation, prolonged hospitalizations, and extended recovery periods. These consequences increase the risk of nosocomial infections, emotional and physical trauma, and higher financial costs. Therefore, timely diagnosis, management by experienced consultants, and close monitoring through multiparametric approaches are essential for achieving better outcomes in cases of Hirschsprung disease. This underscores the importance of ensuring access to appropriate medical services and expertise to facilitate prompt diagnosis and effective management, ultimately improving patient prognosis and reducing associated risks and burdens. Delayed final diagnosis is attributed to the unavailability of healthcare services in nearby localities, which can be overcome by health camps in the interior rural areas.
